# Regional and socioeconomic variations in dietary patterns in a representative sample of young polish females: a cross-sectional study (GEBaHealth project)

**DOI:** 10.1186/s12937-020-00546-8

**Published:** 2020-04-03

**Authors:** Jolanta Czarnocinska, Lidia Wadolowska, Marta Lonnie, Joanna Kowalkowska, Marzena Jezewska-Zychowicz, Ewa Babicz-Zielinska

**Affiliations:** 1 Institute of Human Nutrition and Dietetics, Poznan University of Life Sciences, Wojska Polskiego 28, 60-637 Poznan, Poland; 2 Department of Human Nutrition, University of Warmia and Mazury in Olsztyn, Pl. Cieszynski 1, 10-718 Olsztyn, Poland; 3 Department of Organization and Consumption Economics, Warsaw University of Life Sciences, Nowoursynowska 159 C, 02-776 Warsaw, Poland; 4 Faculty of Physiotherapy and Health Sciences, Gdansk Management College, Pelplinska 7, 80-335 Gdansk, Poland

**Keywords:** Country regions, Socioeconomic status, Dietary patterns, Family, Adolescents, Girls, Females

## Abstract

**Background:**

Associations between dietary patterns (DPs) and socioeconomic correlates among adolescents from emerging economy countries are not fully understood. The study analysed variations in DPs adherence depending on country regions and family socioeconomic status (SES) among Polish females.

**Methods:**

Data from a representative sample (*n* = 1107) of Polish females 13–21-year-old was used. Four DPs were previously identified by principal component analysis. Regions were ranked by Gross Domestic Product. A SES index as an overall measure of family SES was developed. Multiple logistic regression models adjusted for age and body mass index were created.

**Results:**

Higher adherence to ‘Fast-food and sweets’ DP was found in the less affluent (North) region when compared to four other regions (Odds ratio (OR): 1.94 to 1.63). Higher adherence to ‘Fruit and vegetables’ DP was found in more affluent regions when compared to poorer regions: East and North-East (OR 1.71 to 1.81 and 1.69 to 2.23, respectively). Higher adherence to ‘Traditional Polish’ DP was found in 4 out of 5 regions (OR 2.02 to 2.53) when compared to the East. Higher family SES was associated with higher adherence to ‘Fruit and vegetables’ DP (OR 2.06) and lower adherence to ‘Traditional Polish’ DP (OR 0.27).

**Conclusions:**

The study revealed that region’s affluence is strongly reflected in dietary behaviours of young females from a transitioning country. Recognising geographical distribution of dietary patterns within the country and shifting the resources to economically disadvantaged regions might be more effective than current national public health interventions.

## Introduction

Dietary patterns (DPs) are defined as the quantities, proportions, variety, or combination of different foods and drinks in diets, and the frequency with which they are habitually consumed [1]. Some DPs identified across the world can be classified as universal, such as “Western” and “Prudent” patterns, as two polar opposites representing unhealthy and healthy diet [2–5]. Other commonly occurring DPs worldwide, are often labelled as “Traditional”, being specific to the country in which the investigation is being carried [6]. For instance, “Traditional Polish” dietary pattern is usually composed of potatoes, meats, vegetables, cheese, animal fats and sugar [7]. Over the past 30 years, Poland has experienced immense political and cultural changes, transitioning from a socialist to a capitalist economic system. This was reflected in recently reported results in younger populations, where a gradual westernisation of traditional patterns has been observed. The hybrid “Westernised-traditional” [8] or “Westernised-Polish” [9] patterns, in addition to traditional components, tend to be recently complemented by sweets, carbonated beverages, French fries and snacks.

Dietary behaviours can be influenced by various socioeconomic, cultural, demographic and lifestyle factors [9–11]. Socioeconomic status is one of the strongest determinants of health behaviours in both sexes and all age groups [12]. In general, higher socioeconomic status is associated with healthier eating choices, particularly more frequent consumption of fruit and vegetables [12–15]. Interestingly, these associations are stronger in higher income countries, when compared to middle- and lower-income countries [16]. Mayén et al. [13] observed that in lower-middle income countries individuals with higher SES may present mixed dietary behaviours, e.g. have higher adherence to a healthier dietary pattern but also higher energy, cholesterol, and saturated fat intakes. Similar observation was reported in a sample of Brazilian adolescent and young adults (aged 10–39) [17]. A higher adherence to the “ultra-processed food” dietary pattern was positively associated with education and income level [17]. The phenomenon of these dietary disparities, based on the country income, and socioeconomic status still seek a clear explanation and require further investigation.

To date most studies have focused on analysing socioeconomic associations with dietary behaviours using one of two approaches: 1) geographical - by analysing global or local differences in dietary patterns based on the country or area income, e.g. low-income vs. high-income countries [5] or urban vs. rural areas [13, 18, 19] or 2) by analysing individual-level socioeconomic status (education, income, place of residence) and its associations with dietary behaviours in various populations [13, 20, 21]. Some studies compared the two approaches [20, 21], with results suggesting that individual’s socio-economic status may have a stronger effect on diet than the socio-economic characteristics of the living area [21]. We have concluded that it would be interesting to utilise these two approaches and investigate the link with dietary patterns in a representative sample of girls and young women. Only a few studies have investigated the within-country, geographical diversification in DPs characteristics in nationally representative samples [22, 23]. Moreover, to our knowledge, no previous studies have presented results specific to the population of adolescent girls and young women. We hypothesised that the unequal distribution of income in Polish families and the affluence of country regions may be reflected in dietary behaviours of girls and young women.

Poland is classified as an upper-middle income country, with distinct economic inequalities observed between the regions [14]. The Eastern, Northern and North-Western are the most disadvantaged regions with the lowest average monthly gross wages. At the same time, these regions have the highest unemployment rates and the highest monthly percentage of income spent on food (29%), when compared to the most affluent – Central – region (22%) (Supplementary table, S1) [24]. Similarly to other countries, life expectancy is higher for Polish women than men [25]. However, it has been reported that a higher percentage of Polish adolescent girls and young women experience health problems in comparison to boys and young men of the same age. For example, in the age group of 15–29 years old, significantly more females suffer from chronic diseases than males (31.0% vs. 22.6%, respectively) [26]. The national data also revealed that health disparities are observed between the regions, although linear trends with the increasing Gross Domestic Product (GDP) cannot be observed (Supplementary table, S1) [24–26]. Surprisingly, the highest self-reported health status and the lowest prevalence of chronic diseases, smoking and alcohol consumption have been reported in adolescent girls and young women (15–29 years old) from the Eastern region (poorest) (Supplementary table, S1) [26]. The regional mapping of dietary patterns has not been previously investigated.

We believe that the current study can close this remaining evidence gap. The knowledge gained from the proposed dietary mapping strategy could be used by local authorities to plan targeted interventions and distribute funds more efficiently, by allocating the resources to the regions and/or families at need. It can also serve as guidance to other transitioning countries on how to identify nutritional problems and map the areas at highest risk. This study aimed to analyse differences in DPs adherence related to country regions and family socioeconomic status (SES) among young Polish females.

## Methods

### Ethical approval

The study was approved by the Bioethics Committee of the Faculty of Medical Sciences, University of Warmia and Mazury in Olsztyn on June 17, 2010, Resolution No. 20/2010. Informed consent was obtained from adult study participants and from parents/legal guardians of underage girls (< 18 years old). Respecting young participants emerging maturity and independence, each person under 18 years old was involved in the discussions about the research and their verbal assent to participation was obtained. A written assent was not required due to the research posing less than minimal risk.

### Study design

A cross-sectional study was performed using data from a representative sample of Polish females 13–21-year-old enrolled for the Girls Eating Behaviours and Health Study (GEBaHealth) [27, 28]. Data has been collected in 2012 by the interviewers of the Public Opinion Research Centre (CBOS, Warsaw, Poland) at the request of the research team, as a part of the multi-centre scientific project. To collect data, a computer-assisted personal interviewing (CAPI) technique, instead of a printed version of a questionnaire, was applied. For questions with many optional responses, the interview has been supported with so-called ‘show cards’ containing responses to choose from. The interviewers were trained in dietary data collection; first – leaders have been trained by an experienced researcher, then leaders trained other interviewers from their team. Self-declared data regarding socioeconomic status, dietary habits, and also weight and height were collected [27, 28].

### Participants

The inclusion criteria were female gender, age (born between 1991 and 1999) and place of residence (Poland). Extensive details of recruitment and sample size estimation have been previously reported [27, 28]. Briefly, based on a pilot study we calculated that 1000 participants would be a sufficient number of participants for our study. Expecting a 50% non-response rate and taking into account potential missing data, a total of 2104 females from the Universal Electronic System of Population Register (PESEL) were randomly selected to contact them. The response rate was 52.6%. To ensure the sample is representative for the Polish population three-phase random sampling was used. The sample was stratified by age, place of residence and region [25]. Sample weights were calculated to account for non-response (997 respondents). Finally, 1107 participants aged 13–21 years take part in the study. Flow chart of sample collection is presented in the supplementary material Fig. (S1).

### Country regions

**Fig. 1:**
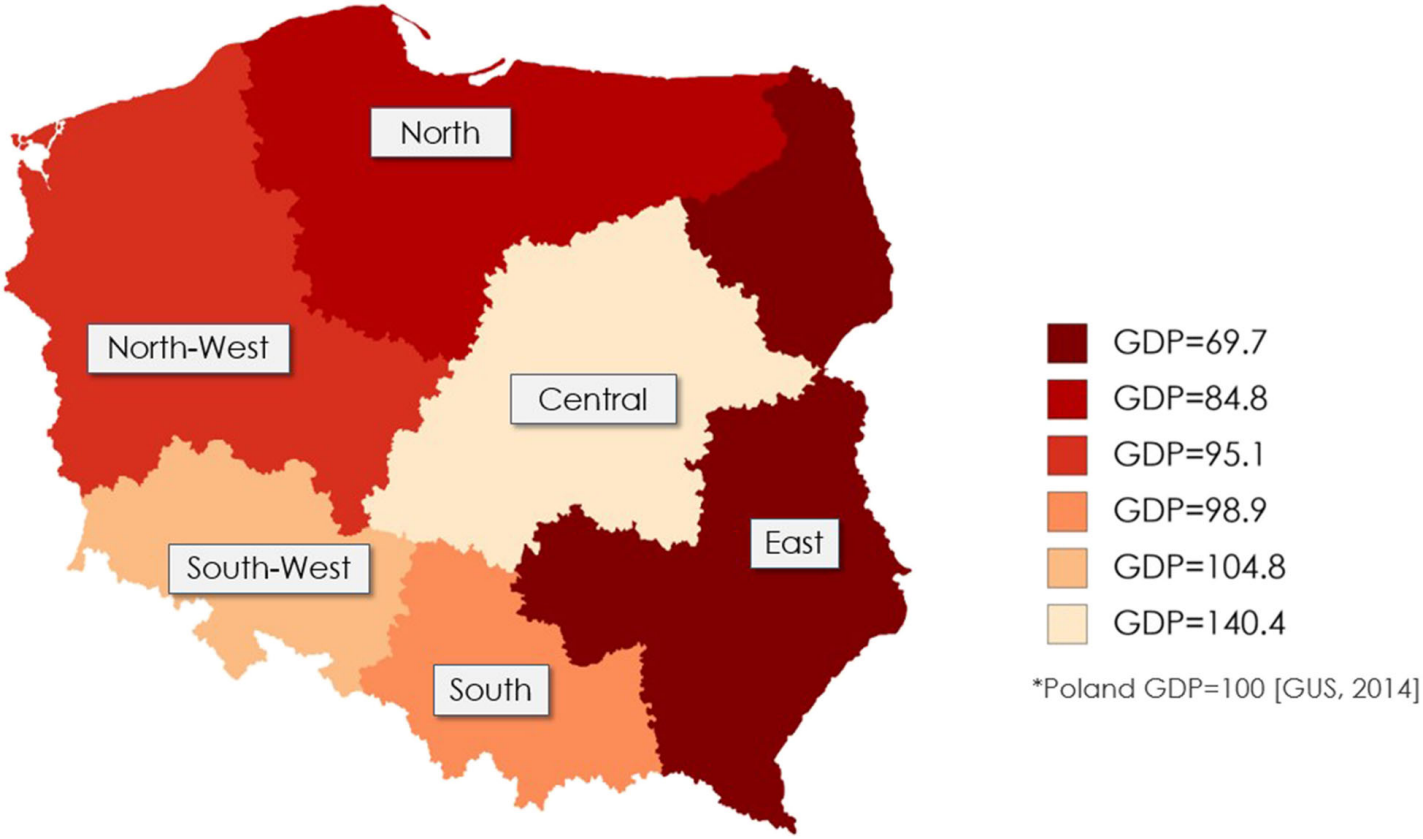
Division of Poland by regions: darker colours represent regions with lower Gross Domestic Product (GDP).

In line with the Polish database of Central Statistical Office, six regions of the Poland territory were considered. Regions were ranked by Gross Domestic Product (GDP) from less to more wealthy (Poland GDP = 100) as follows: East GDP = 69.7, North GDP = 84.8, North-West GDP = 95.1, South GDP = 98.8, South-West GDP = 104.8, Central GDP = 140.4 (Fig. 1) [24]. More data related to the economic characteristics of the regions are presented in the supplementary material (Table S1).

### Family socioeconomic status (SES)

To measure a family SES, we developed previously a SES index as an overall measure [27, 28]. While collecting socioeconomic data, we considered six single factors reflecting both objective and subjective SES measures: mother’s education, father’s education, economic status, description of household, place of residence and number of children (< 18 years old) in a family. All factors had response categories with numerical values (points) assigned as follows:

- mother’s education – three responses to choose from: primary/lower secondary (1 point), upper secondary (2 points), higher (3 points);
- father’s education – three responses to choose from: primary/lower secondary (1 point), upper secondary (2 points), higher (3 points);
- economic status – three responses to choose from: below average (1 point), average (2 points), above average (3 points);
- description of the household overall situation – five responses (all with detailed explanation) to choose from: we live poorly – we do not have enough money for basic needs (1 point), we live modestly – we have to be very careful with our daily budget (2 points), we live relatively thriftily – we have enough money for our daily needs, but we need to budget for bigger purchases (3 points), we live well – we have enough money for our needs without particular budgeting (4 points), we live very well – we can afford some luxury (5 points);
- place of residence – three responses to choose from: village (1 point), town (2 points), city (≥100,000 inhabitants; 3 points);
- number of children (< 18 years old) in a family – respondents reported the number of minors in their family, and four categories were established: 6 or more children (1 point), 4–5 children (2 points), 2–3 children (3 points), 1 child (4 points).

For each participant, the SES index was calculated as the sum of numerical values assigned to each category of single SES variables. Due to the uneven contribution of single SES factors, the variables were standardised. We used Cronbach’s alpha to measure the internal consistency of input data [29] and two of six SES factors (place of residence and number of children in a family) were excluded. Finally, the SES index consisted of four single factors: (i) two objective measures (mother’s education, father’s education and (ii) two subjective evaluations (economic status, description of the household). The Cronbach’s alpha for a set of four SES factors was 0.689. Based on the tertile distribution of SES index, respondents were categorised into three categories: low, medium and high SES.

### Dietary patterns (DPs)

Dietary patterns were previously derived by principal components analysis (PCA). With this technique, new variables are found (dietary patterns) which are linear functions of originally observed variables (food items) in the dataset [30, 31]. Dietary data were collected using three short-form food frequency questionnaires: Food Intake Variety Questionnaire (FIVeQ) [32], Block Screening Questionnaire for Fruit/Vegetable/Fibre Intake (BSQFVF) [33] and Block Screening Questionnaire for Fat Intake (BSQF) [33]. We modified and adjusted both of the Block’s questionnaires to Polish diet [27] while the FIVeQ was assessed as a reliable tool to measure a variety of food intake of Poles [32]. In summary, all three questionnaires provided a total of 30 food items that were included into the PCA to derive dietary patterns. Before the PCA, data was standardised. All the variables were scaled to achieve mean equal 0 and standard deviation equal 1 and to bring all of them to the same range and/or variability. We considered eigenvalues of at least 1.00, scree plot and variance explained. Items that had factor loading >|0.40| were used [29] to identify and name each DP. A varimax normalised rotation was used in order to extract non-correlated factors and obtain large variance explained.

A detailed description of identified DPs was reported elsewhere [28]. Briefly, four patterns explaining 33.9% (14.5, 9.0, 5.6 and 4.8%, respectively) were retained. Positive loadings of the food item indicate its high correlation with the corresponding dietary pattern, whereas negative loadings suggest its inverse correlation. Patterns were labelled qualitatively, according to the combinations of foods with highest factor loadings [31]. The first dietary pattern was labelled ‘Traditional Polish’ and was correlated with: white bread frequency consumption (factor-loading 0.65), meats/fish/eggs intake variety (0.60), potato frequency consumption (0.52), red meat frequency consumption (0.51), margarine or butter frequency consumption (0.45), fried chicken frequency consumption (0.42), fat intake variety (0.40), wholegrain bread frequency consumption (− 0.48). The second dietary pattern was labelled ‘Fruit and vegetables’ and was correlated with intake variety (0.60), green salad frequency consumption (0.57), fruit frequency consumption (0.55), prepared vegetable frequency consumption (0.55), fruit intake variety (0.54) and bean frequency consumption (0.45). The third dietary pattern was labelled ‘Fast food and sweets’ and was correlated with French fries or potato chips or corn chips or popcorn frequency consumption (0.71), hamburger or cheeseburger frequency consumption (0.60), ice cream frequency consumption (0.52), doughnut, pastry, cake or cookie frequency consumption (0.50), sweets and snacks intake variety (0.47) and salad dressing or mayonnaise (not diet) frequency consumption (0.42). The fourth dietary pattern was labelled ‘Dairy and fats’ and was correlated with cereal and potato intake variety (0.56), dairy product intake variety (0.54), cheese or cheese spread frequency consumption (0.54), whole milk frequency consumption (0.49), margarine or butter frequency consumption (0.45), and fat intake variety (0.43) [28]. Next, based on tertile distribution, participants were divided into three categories within each DP as follows: bottom, middle, upper tertile. Main findings are presented in Table 1 and supplementary materials (Table S3).

**Table 1:** Description of PCA-derived dietary patterns [26]

Dietary patterns	Components of DPs (factor loadings) related to:	
	Food consumption frequency of ^a^	Food intake variety of ^b^
‘Traditional Polish’	White bread (0.65), potatoes (0.52), red meats (0.51), margarine or butter (0.45), fried chicken (0.42), wholegrain bread (−0.48);	Meats/fish/eggs (0.60), fats (0.40);
‘Fruit and vegetables’	Green salad (0.57), fruit (0.55), prepared vegetables (0.55), beans (0.45);	Vegetables (0.60), fruit (0.54);
‘Fast-food and sweets’	French fries or potato chips or corn chips or popcorn (0.71), hamburgers or cheeseburgers (0.60), ice cream (0.52), doughnuts or pastries or cake or cookies (0.50), salad dressings or mayonnaise (not diet) (0.42);	Sweets and snacks (0.47);
‘Dairy and fats’	Cheese or cheese spread (0.54), whole milk (0.49), margarine or butter (0.45);	Cereals and potatoes (0.56), dairy products (0.54), fats (0.43);

Notes: ^a^ Food consumption frequency (range 0–4 points) measured: (A) with BSQFVF for 9 food items: fruit or vegetable juices, fruit, green salad, potatoes, beans, processed vegetables, cereals, wholegrain bread, white bread; and expressed in frequencies (with assigned points): ‘less than once per week’ (0 points), ‘about once per week’ (1 point), ‘2–3 times per week’ (2 points), ‘4–6 times per week’ (3 points), ‘daily’ (4 points), (B) with BSQF for 13 food items: hamburgers or cheeseburgers, red meats, fried chicken, hot dogs, luncheon meats/bacon/sausages, salad dressings/mayonnaise, margarine/butter, eggs, cheese/cheese spread, whole milk, French fries/potato chips/corn chips/popcorn, ice cream, doughnuts/pastries/cakes/cookies; and expressed in frequencies (with assigned points): ‘less than once per month’ (0 points), ‘2–3 times per month’ (1 point), ‘1–2 times per week’ (2 points), ‘3–4 times per week’ (3 points), ‘5 times per week and more’ (4 points) [27, 28, 33]; ^b^ Food intake variety (with ranges from 0 to 4 to 0–14 foods/week) measured as a number food items consumed per week within 8 food groups (maximum food items): cereals and potatoes (6 items), dairy products (4 items), meats, fish and eggs (12 items), vegetables (14 items), fruit (8 items), fats (6 items), sweets and snacks (4 items), beverages (6 items, without alcohol) [32]

### Confounding variables

Two variables were considered as potential confounders: age and body mass index (BMI). The selection of moderators was evidence-based: age is a key variable influencing dietary behaviours while BMI is a convenient, practical marker of energy-balance [34, 35]. These variables were described in detail elsewhere [27]. In brief, we calculated the participants’ age with the accuracy of a month. To calculate BMI, we used self-reported weight and height data. Next, we used the regression equations to correct for potential biases when self-reported data is used [36]. The regression equations have been developed previously using multiple regression and based on self-reported and measured data on body weight and height collected in 916 Polish boys and girls aged 13–20 years [36]. Two equations developed for girls and listed below were used:

1 $$ MW= 0.9740\ x\  SRW+ 0.1210\ x\ A\pm 3.1251 $$

2 $$ MH= 0.9428\ x\  SRH+ 9.4831\pm 2.5405 $$

#### Abbreviations: MW – measured weight (kg), SRW – self-reported weight (kg), MH – measured height (cm), SRH – self-reported height (cm), a – age)

BMI was calculated with corrected data on weight and height. BMI was categorised into six categories according to International Obesity Task Force (IOTF) standards [37], for girls 13–18 years old according to age-sex-specific BMI cut-offs, for girls > 18 years old according to cut-offs for girls at age 18) [37] as follows: < 16.0 kg/m^2^ (thinnest grade 3), 16.0–16.9 kg/m^2^ (thinnest grade 2), 17.0–18.4 kg/m^2^ (thinnest grade 1), 18.5–24.9 kg/m^2^ (normal weight), 25.0–29.9 kg/m^2^ (overweight) and ≥ 30 kg/m^2^ (obesity).

### Statistical analysis

Categorical variables were presented as a sample percentage (%), and continuous variable as means with standard deviation (SD). To check the distribution of continuous variable (age) Kolmogorov-Smirnov test was used. This variable was not normally distributed, and a non-parametric test was used to compare the mean values (the Kruskal-Wallis test). For categorical variables, the differences between groups were verified by chi^2^ test. All data were adjusted for sample weights to maintain the representativeness. To verify the associations between DPs and variables under study, logistic regression analysis was used. The categorical variables with at least three categories were recoded into separate binary variables (dummy coding). Then, the odds ratios (ORs) and 95%CIs were calculated. Wald’s statistics were used to assess the significance of ORs. Subjects from the bottom tertiles of each DP were used as a reference group (OR = 1.00). Two models were created: crude and adjusted for age (a continuous variable) and age-sex-specific BMI (a categorical variable) as they were independently associated with the dietary patterns (Table 2). For all tests *p*-value < 0.05 was considered as significant. Statistical analyses were carried out using STATISTICA software (version 10.0 PL; StatSoft Inc., USA, Tulsa; StatSoft Polska, Krakow).

**Table 2:** Sample distribution (%) or means (with standard deviation) in relation to country region, family socioeconomic status (SES), age and body mass index (BMI) by tertiles of dietary patterns

Variables	Total	‘Traditional Polish’				‘Fruit and vegetables’				‘Fast food and sweets’				‘Dairy and fats’			
		Bottom	Middle	Upper	*P*	Bottom	Middle	Upper	*P*	Bottom	Middle	Upper	*P*	Bottom	Middle	Upper	*P*
Number of respondents	1107	367	364	376		364	367	376		365	365	377		366	365	376	
Age (years)	17.3 (2.6)	17.6 (2.5)	17.1 (2.6)	17.1 (2.6)	0.0032	17.0 (2.6)	17.4 (2.5)	17.4 (2.6)	0.0771	17.5 (2.6)	17.2 (2.6)	17.2 (2.6)	0.3652	17.8 (2.5)	17.1 (2.6)	17.0 (2.6)	< 0.0001
Region ^a^					0.0170				0.0772				0.4819				0.3621
East	18.1	40.1	37.0	22.9		37.8	34.3	28.0		33.0	33.8	33.2		34.6	35.3	30.1	
North	14.9	36.5	25.9	37.6		33.2	32.0	34.8		25.4	32.8	41.8		25.3	36.6	38.1	
North-West	16.5	29.3	32.9	37.8		38.6	35.5	25.9		35.0	34.1	30.9		35.1	32.2	32.7	
South	21.3	26.9	35.6	37.5		27.8	34.7	37.5		37.3	30.9	31.8		31.7	31.5	36.8	
South-West	9.5	37.2	31.0	31.7		30.5	27.7	41.8		31.1	36.9	32.0		29.8	34.3	35.9	
Central	19.7	32.0	32.5	35.6		30.1	32.0	37.9		33.2	32.0	34.8		38.8	29.6	31.6	
SES index ^b^					< 0.0001				0.0007				0.7716				0.4559
Low	36.2	22.0	34.4	43.6		40.4	32.2	27.4		32.1	34.9	33.0		33.5	33.9	32.5	
average	30.6	34.0	30.2	35.8		30.0	33.1	36.9		33.9	33.1	33.0		30.0	35.1	34.9	
High	33.2	44.3	33.8	21.9		27.4	34.3	38.4		33.1	30.8	36.0		35.4	29.9	34.7	
**Components of SES index**																	
Mother’s education					< 0.0001				0.0473				0.0118				0.0846
primary/lower secondary	39.8	24.1	33.1	42.8		37.5	31.7	30.8		27.3	36.8	35.9		32.9	34.2	32.9	
upper secondary	43.8	36.6	31.6	31.9		31.3	34.0	34.7		37.5	29.0	33.5		30.4	32.4	37.3	
higher	16.4	45.7	35.9	18.4		25.8	34.6	39.6		34.6	34.5	30.9		40.6	31.4	28.0	
Father’s education					< 0.0001				< 0.0001				0.0080				0.0487
primary/lower secondary	52.4	25.8	33.3	40.9		38.8	32.9	28.4		28.3	36.2	35.5		32.6	35.1	32.4	
upper secondary	35.1	39.5	31.7	28.7		29.1	32.7	38.2		38.3	28.3	33.4		30.4	31.9	37.7	
Higher	12.5	45.5	34.6	19.9		18.9	35.7	45.5		37.6	33.1	29.3		42.6	27.0	30.4	
Economic status					0.0134				0.0204				0.1319				0.0656
below average	3.9	19.0	31.2	49.8		47.3	21.3	31.4		25.1	41.8	33.1		25.3	50.0	24.7	
average	80.0	32.6	32.4	35.0		33.4	32.0	34.7		34.3	33.1	32.6		32.4	32.3	35.3	
above average	16.1	39.1	35.6	25.3		27.1	41.9	31.0		28.5	30.3	41.3		38.2	32.1	29.8	
Description of household – we live:					< 0.0001				0.1576				0.1469				0.3394
modestly ^c^	8.2	16.0	38.1	45.9		41.6	26.1	32.3		35.7	35.7	28.6		24.0	33.8	42.2	
relatively thriftily	49.6	30.0	32.5	37.5		33.8	34.3	31.9		35.9	31.8	32.3		34.0	33.1	32.9	
well ^d^	42.2	40.0	32.4	27.6		30.1	33.2	36.6		29.1	33.9	37.0		33.8	32.6	33.6	
**Weight status**																	
BMI categories ^e^					0.1014				0.0059				0.0033				0.0477
thinnest grade 3	0.0	0.0	0.0	0.0		0.0	0.0	0.0		0.0	0.0	0.0		0.0	0.0	0.0	
thinnest grade 2	0.5	34.4	39.6	26.0		34.8	30.3	34.9		9.5	48.4	42.1		69.7	0.0	30.3	
thinnest grade 1	9.7	22.7	31.8	45.5		35.1	30.9	34.0		30.3	21.2	48.5		24.5	40.5	35.0	
normal weight	77.7	34.8	32.5	32.7		34.9	33.8	31.3		31.6	34.5	33.9		31.9	32.8	35.3	
overweight	10.5	32.6	37.6	29.8		19.2	31.1	49.7		43.9	31.8	24.3		43.4	29.0	27.6	
obesity	1.6	15.8	34.1	50.1		13.2	41.7	45.1		44.3	37.0	18.7		39.8	39.0	21.2	

Notes: Sample size may vary in each variables due to missing data; All data adjusted for sample weights; ^a^ Regions of Poland ranked by Gross Domestic Product (GDP) from less to more wealthy: East GDP = 69.7, North GDP = 84.8, North-West GDP = 95.1, South GDP = 98.8, South-West GDP = 104.8, Central GDP = 140.4, Poland GDP = 100 [24]; ^b^ SES index categories based on tertile distribution; ^c^ ‘Modestly’ category was developed by combining two answers: ‘we live poorly’ and ‘we live modestly’; ^d^ ‘Well’ category was developed by combining two answers: ‘we live well’ and ‘we live very well’; ^e^ BMI was categorised according to IOTF standards [37], for girls 13–18 years old according to age-sex-specific BMI cut-offs, for girls > 18 years old according to cut-offs for girls at age 18) [37] as follows: < 16.0 kg/m^2^ (thinnest grade 3), 16.0–16.9 kg/m^2^ (thinnest grade 2), 17.0–18.4 kg/m^2^ (thinnest grade 1), 18.5–24.9 kg/m^2^ (normal weight), 25.0–29.9 kg/m^2^ (overweight) and ≥ 30 kg/m^2^ (obesity); *P* – significance level of the following tests: chi^2^ test for categorical variables or Kruskal–Wallis test for continuous variable

## Results

Participants’ distribution by regions was significantly different by tertiles of ‘Traditional Polish’ DP while participants’ distribution by SES index was significantly different by tertiles of ‘Traditional Polish’ and ‘Fruit & vegetables’ DPs (Table 2).

### Variations in DPs by regions

Higher adherence to ‘Fast-food and sweets’ pattern was found in the North region when compared to four others as reference: the East (OR 1.94, 95% CI 1.20–3.15), the North-West (OR 1.93, 95% CI 1.18–3.17), the South (OR 2.10, 95%CI 1.31–3.36), and the Central (OR 1.63, 95%CI 1.01–2.63) (Table 3; Supplementary file: Fig. S2). Higher adherence to ‘Fruit and vegetables’ pattern was found in 2 out of 5 regions (the South OR 1.71; 95% CI 1.10–2.67; the South-West OR 1.81; 95% CI 1.06–3.11) when compared to the East as reference, and also in 4 out of 5 regions (the North OR 1.64, 95%CI 1.00–2.69; the South OR 2.23, 95% CI 1.38–3.61; the South-West OR 2.10, 95% CI 1.20–3.65; the Central OR 1.69, 95% CI 1.05–2.73) when compared to the North-West as reference. Higher adherence to ‘Traditional Polish’ was found in 4 out of 5 regions (the North OR 2.02, 95%CI 1.26–3.25; the North-West OR 2.07, 95% CI 1.28–3.32; the South OR 2.53, 95% CI 1.60–4.01; the Central OR 2.24; 95% CI 1.41–3.58) when compared to the East (the poorest).

**Table 3:** Adjusted ^a^ odds ratios (95% Confidence intervals) for country region and family socioeconomic status (SES) by tertiles of dietary patterns

Variables	Bottom tertile	‘Traditional Polish’		‘Fruit and vegetables’		‘Fast food and sweets’		‘Dairy and fats’	
		Upper tertile	*P*	Upper tertile	*P*	Upper tertile	*P*	Upper tertile	*P*
Region ^b^									
East	Ref.	1.00		1.00		1.00		1.00	
North		**2.02** (1.26–3.25)	0.0036	1.35 (0.84–2.15)	0.2136	**1.94** (1.20–3.15)	0.0070	**2.05** (1.25–3.36)	0.0044
North-West		**2.07** (1.28–3.32)	0.0027	0.79 (0.49–1.27)	0.3275	1.04 (0.65–1.66)	0.8815	1.16 (0.73–1.85)	0.5342
South		**2.53** (1.60–4.01)	< 0.0001	**1.71** (1.10–2.67)	0.0178	0.98 (0.63–1.53)	0.9424	1.41 (0.91–2.19)	0.1214
South-West		1.42 (0.82–2.46)	0.2139	**1.81** (1.06–3.11)	0.0290	1.21 (0.70–2.10)	0.4856	1.54 (0.88–2.69)	0.1321
Central		**2.24** (1.41–3.58)	0.0007	1.44 (0.92–2.25)	0.1131	1.19 (0.76–1.87)	0.4441	1.03 (0.66–1.61)	0.9046
North	Ref.	1.00		1.00		1.00		1.00	
East		**0.50** (0.31–0.80)	0.0041	0.75 (0.47–1.19)	0.2172	**0.52** (0.32–0.84)	0.0070	**0.50** (0.31–0.82)	0.0056
North-West		1.10 (0.68–1.77)	0.6915	0.61 (0.37–1.00)	0.0495	**0.52** (0.32–0.85)	0.0087	**0.60** (0.36–0.99)	0.0446
South		1.27 (0.80–2.02)	0.3087	1.30 (0.82–2.07)	0.2601	**0.48** (0.30–0.77)	0.0021	0.73 (0.46–1.18)	0.1958
South-West		0.79 (0.46–1.36)	0.3851	1.30 (0.76–2.24)	0.3341	0.63 (0.36–1.11)	0.1075	0.75 (0.42–1.36)	0.3440
Central		1.10 (0.68–1.76)	0.7011	1.04 (0.65–1.67)	0.8649	**0.61** (0.38–0.99)	0.0445	**0.54** (0.33–0.88)	0.0123
North-West	Ref.	1.00		1.00		1.00		1.00	
East		**0.48** (0.30–0.78)	0.0027	1.28 (0.79–2.07)	0.3177	0.96 (0.60–1.54)	0.8808	0.86 (0.54–1.37)	0.5233
North		0.91 (0.57–1.46)	0.6915	**1.64** (1.00–2.69)	0.0496	**1.93** (1.18–3.17)	0.0087	**1.67** (1.01–2.77)	0.0446
South		1.14 (0.72–1.80)	0.5761	**2.23** (1.38–3.61)	0.0011	0.92 (0.58–1.46)	0.7245	1.21 (0.77–1.90)	0.4016
South-West		0.74 (0.43–1.27)	0.2756	**2.10** (1.20–3.65)	0.0085	1.24 (0.71–2.15)	0.4491	1.35 (0.77–2.37)	0.2910
Central		1.01 (0.64–1.61)	0.9553	**1.69** (1.05–2.73)	0.0300	1.20 (0.75–1.93)	0.4379	0.89 (0.56–1.41)	0.6066
South	Ref.	1.00		1.00		1.00		1.00	
East		**0.40** (0.25–0.63)	< 0.0001	**0.59** (0.38–0.92)	0.0184	1.02 (0.66–1.58)	0.9315	0.73 (0.47–1.12)	0.1499
North		0.79 (0.49–1.25)	0.3088	0.77 (0.48–1.22)	0.2600	**2.10** (1.31–3.36)	0.0021	1.36 (0.85–2.19)	0.1957
North-West		0.88 (0.55–1.39)	0.5763	**0.45** (0.28–0.73)	0.0011	1.09 (0.69–1.72)	0.7247	0.83 (0.53–1.29)	0.4016
South-West		0.63 (0.37–1.07)	0.0871	0.98 (0.57–1.69)	0.9513	1.35 (0.79–2.31)	0.2685	1.04 (0.61–1.78)	0.8762
Central		0.85 (0.54–1.34)	0.4849	0.80 (0.51–1.25)	0.3215	1.26 (0.81–1.97)	0.2989	0.74 (0.48–1.14)	0.1683
South-West	Ref.	1.00		1.00		1.00		1.00	
East		0.71 (0.41–1.23)	0.2140	**0.56** (0.33–0.97)	0.0369	0.83 (0.48–1.44)	0.5012	0.66 (0.38–1.16)	0.1494
North		1.27 (0.74–2.19)	0.3850	0.77 (0.45–1.32)	0.3342	1.59 (0.90–2.79)	0.1068	1.33 (0.74–2.39)	0.3440
North-West		1.35 (0.79–2.31)	0.2757	**0.48** (0.27–0.83)	0.0085	0.81 (0.47–1.40)	0.4491	0.74 (0.42–1.30)	0.2910
South		1.58 (0.93–2.69)	0.0863	1.02 (0.60–1.72)	0.9501	0.74 (0.43–1.26)	0.2685	0.96 (0.56–1.64)	0.8767
Central		1.50 (0.87–2.60)	0.1452	0.76 (0.45–1.30)	0.3186	1.00 (0.67–1.49)	0.9872	0.67 (0.39–1.16)	0.1516
Central	Ref.	1.00		1.00		1.00		1.00	
East		**0.45** (0.28–0.72)	0.0008	0.69 (0.44–1.09)	0.1098	0.84 (0.54–1.32)	0.4528	0.98 (0.62–1.53)	0.9238
North		0.91 (0.57–1.47)	0.7017	0.96 (0.60–1.53)	0.8644	**1.63** (1.01–2.63)	0.0445	**1.86** (1.14–3.02)	0.0123
North-West		0.99 (0.60–1.62)	0.9580	**0.59** (0.37–0.95)	0.0300	0.83 (0.52–1.33)	0.4379	1.13 (0.71–1.79)	0.6066
South		1.18 (0.75–1.85)	0.4850	1.25 (0.80–1.95)	0.3215	0.79 (0.51–1.23)	0.2989	1.36 (0.88–2.09)	0.1682
South-West		0.67 (0.39–1.15)	0.1452	1.31 (0.77–2.22)	0.3186	1.00 (0.56–1.80)	0.9911	1.48 (0.86–2.55)	0.1516
SES index ^c^									
low	Ref.	1.00		1.00		1.00		1.00	
average		**0.58** (0.41–0.81)	0.0014	**1.88** (1.34–2.64)	0.0003	0.87 (0.62–1.22)	0.4263	1.20 (0.85–1.69)	0.2935
high		**0.27** (0.19–0.39)	< 0.0001	**2.06** (1.47–2.88)	< 0.0001	0.94 (0.68–1.31)	0.7150	0.88 (0.63–1.21)	0.4231

Notes: Sample size may vary in each variables due to missing data; All data adjusted for sample weights; ^a^ Odds ratios adjusted for age (years) and BMI (as categorical variable according to IOTF standards [37]), for girls 13–18 years old according to age-sex-specific BMI cut-offs, for girls > 18 years old according to cut-offs for girls at age 18); ^b^ Regions of Poland ranked by Gross Domestic Product (GDP) from less to more wealthy: East GDP = 69.7, North GDP = 84.8, North-West GDP = 95.1, South GDP = 98.8, South-West GDP = 104.8, Central GDP = 140.4, Poland GDP = 100 [24]; ^c^ SES index categories based on tertile distribution; *P* – significance level of the Wald’s test. Significant odds ratios are bolded

Higher adherence to ‘Dairy and fats’ pattern was found in the North region when compared to three others as reference: the East (OR 2.05, 95% CI 1.25–3.36), the North-West (OR 1.67, 95% CI 1.01–2.77), and the Central (OR 1.86, 95% CI 1.14–3.02). Similar significant associations in crude models were shown for all DPs (Supplementary file: Table S3).

### Variations in DPs by family SES

Higher adherence to ‘Fruit and vegetables’ pattern was found in high SES index (OR 2.06; 95% CI 1.47–2.88) when compared to low SES index as reference. Lower adherence to ‘Traditional Polish’ was found in high SES index (OR 0.27; 95%CI 0.19–0.39) when compared to low SES index. The same significant associations in crude models were shown for all DPs (Supplementary file: Table S3).

## Discussion

The study revealed that the higher adherence to the ‘Fruit and vegetables’ pattern was observed in the more affluent regions of Poland (the Central, the South-West, the South) and the second poorest region (the North) when compared to others, and also in families with high SES index when compared to the low SES index. The higher adherence to the ‘Fast-food and sweets’ pattern was only observed among participants from the second poorest region (the North). ‘Traditional Polish’ pattern did not appear to be region-specific, however it was less common among girls from the families with higher socioeconomic status. In the most economically disadvantaged region (the East) the lower adherence to all four dietary patterns was shown. The analysis of country’s regions revealed more associations with dietary patterns than the analysis of family socioeconomic status in a representative sample of Polish girls in young women, suggesting country’s regions can be a valid, perhaps more sensitive measure used to identify areas at need of dietary interventions.

Our findings are in line with previous reports, confirming that family socioeconomic status and the affluence of the place of residence are both important factors in terms of fruit and vegetables intake. The PURE study analysed the frequency of fruit and vegetables consumption among adults in 18 countries (including Poland) and found that the lowest intake was observed in low-income countries (2.14 serving/day), while the highest in high-income countries (5.42 serving/day) [14]. It was concluded that the affordability of fruit and vegetables was the main contributing factor to the intake frequency [14]. Although the results of our study were not unexpected, the current study provides the first representative data on region-specific dietary behaviours of Polish adolescent girls and young women. Our previous findings from the GEBaHealth study revealed that higher adherence to the “Fruit and vegetables” pattern was observed among girls with positive attitudes towards health, those who use dietary restrictions towards unhealthy foods and with the higher level of physical activity at work or school [27, 38, 39]. All these traits can be mediated by a higher family socioeconomic status and region’s progressive, health-promoting infrastructure which allows more opportunities to physical activity (e.g. active commuting, better school sports facilities) [40, 41], and easier access to fresh, affordable fruit and vegetables [42]. It has been shown that in contrary to affluent regions, access to fruit and vegetables is limited in rural or less urbanised regions, often being called ‘food deserts’ [43–45]. Perhaps, the increase in farmers’ markets or closer proximity to supermarkets (with more competitive pricing) could improve access to healthy foods in the most disadvantaged regions of Poland. The improvement in fruit and vegetable intake could also be facilitated through policy regulations or subsidising families with the lowest income, e.g. by providing fruit and vegetable vouchers [46].

Unhealthy dietary behaviours were observed in the Northern region of Poland, being the area with the second-lowest gross domestic product. Interestingly, the analysis of family socioeconomic status did not detect this association. Our findings suggest that dietary behaviours of girls from this region may contribute to their health. According to the national data, the Northern region has the highest percentage of chronic diseases among females aged 15–29 with (35.7%); compared to the national average of 31% (Supplementary data, Table S1) [26]. High adherence to ‘Fast-foods and sweets’ dietary pattern in the Northern region confirms previous findings that poor-quality diet is more common in economically disadvantaged communities [47–49]. Westernised diet characterised by frequent intake of processed foods rich in fat and/or sugar and low in fibre can be attractive for two reasons. First, due to its relatively low cost [50]. Secondly, due to the perception of these foods acquired in childhood and adolescence. During the transition period, Poland suddenly became exposed to western culture, with the western lifestyle being strongly promoted in the media and advertising [51]. Initially, dining at fast-food restaurants was perceived as a sign of prosperity, available only to more affluent families. Within time, the cost of fast-foods and sweets became more affordable, however, the preference for convenient foods and a feeling of reward may have persisted and tracked to adulthood. The increased attractiveness of western products and fast-foods in Poland during the transition period is not country-specific and has been reported in many emerging economies worldwide [52–54]. The adherence to the ‘Fast-foods and sweets’ pattern was not observed in the poorest (Eastern) region, which could be explained by the lowest urbanisation of this region, hence the access to fast-food restaurants could have been still limited. This is an interesting observation, suggesting that the fast-food consumption might be a direct or indirect moderator of health status and stimulant use. According to the national data, the Eastern region, despite low frequency of fruit and vegetable intake, had the highest percentage of young females (aged 15–29 years) with good or very good self-reported health (91%), and the lowest percentage of chronic diseases (22.2%), smoking (13.3%) and alcohol consumption (7.9%), comparing to the national average in this population group (89.1, 31, 16 and 10% respectively) (Supplementary material, Table S1) [26]. Our own data have also confirmed that girls from the Eastern region had the lowest rate of overweight (5.3%), in comparison to the North-West, North and South-West regions (13.8, 13.5 and 13.4% respectively) (Supplementary material, Table S4). The adherence to ‘Fast-foods and sweets’ pattern was not strong in regions with higher income. Perhaps, the interest in fast-foods and sweets has already peaked in more affluent and better educated families, resulting in the gradual shift towards healthier dietary choices [55, 56].

It can be presumed that region’s affluence is not a contributing factor to the traditional way of eating among Polish adolescent girls and young women. Although, the analysis of family socioeconomic status revealed, that this pattern was less common in girls with the higher SES. When compared to the East, high adherence to the ‘Traditional Polish’ dietary pattern was observed across the country, except the South-West region (the second richest region). It is most likely that the historical background of the East and South-West regions was a key factor since both regions were strongly influenced in the past by Russian and German cultures, respectively. Moreover, considering the young age of study participants it is very likely, that the girls and young females were still relying on meals prepared by the parents, who are still more familiar with cooking traditional Polish cuisine. Interestingly, the higher adherence to ‘Dairy and fats’ dietary pattern was observed only in the Northern region, which can be explained by the cultural influences of Baltic and Scandinavian countries. The importance of country historical and cultural background has been previously reported. Krieger et al. [23] found that language region (German, French or Swiss) was the main determinant of dietary patterns in a representative sample of Swiss adults. Considering that Switzerland is a high-income country the affordability of healthy foods was not such a strong determinant of dietary behaviours in this sample. Despite cultural influences, it can be presumed, that in Poland, the price and accessibility still appear to be one of the crucial factors determining eating choices, and it can be assumed that the cultural background was only of mild importance. The income and food price have been previously recognised as key determinants of dietary intake in both rich and poor countries [57]. Hence, increasing the affordability of health-promoting foods should be considered a key strategy for national intervention programs.

### Strengths and limitations

The main strength of this study is a large, nationally representative sample of 1107 adolescent girls and young women. Although our findings are specific to the dietary behaviours of the Polish females, it provides a valuable insight into the importance of region-specific analysis of health behaviours within the country, and within a specific population group. It has to be acknowledged, that the frequency of fruit and vegetable intake is only a single indicator of a healthy lifestyle and it may be complemented by other behaviours in young females – favourable or unfavourable to health, e.g. dietary restraint practices [39, 58]. Our previous studies showed that girls and young females may engage in explicit health behaviours [38, 39], driven by psychological factors and are more prone to favour body image over health [59–61]. Hence a careful interpretation needs to be applied. The evidence suggests, that dietary patterns should be studied with gender and age stratification [62], hence our results should not be generalised to males and other age groups. The cross-sectional design allowed only for identifying the regional variations in dietary patterns, and the associations with health status of girls in each region remain unclear. However, in our previous work we have found that the positive attitudes towards health were associated with higher adherence to ‘Fruit and vegetable’ pattern, while negative attitudes were associated with higher adherence to ‘Traditional Polish’ and ‘Fast foods and sweets’ patterns [38].

Next, the subjective evaluations of an economic situation of the family were used instead of objective measure such as an income. The income can be a particularly sensitive question, potentially difficult to answer and causing discontent, which in consequence can lead to greater non-response rates than other SES measures [63]. Instead, it was decided to use two subjective evaluations, which were simple, tailored to Polish realities and easy to provide a reliable answers by females aged 13–21 years who may not know their family’s income or could feel the discomfort associated with the direct question of income. Lastly, since our study relied on self-reported data, there is a possibility of social desirability bias, particularly in terms of reported body weight and food intake. It has been shown, that young people tend to overestimate the consumption of foods perceived as healthy (e.g. fruit and vegetables) and underreport the consumption of unhealthy foods (e.g. fast foods and sweets) [64]. To address the risk of potential dietary misreporting, the results were adjusted for external predictors such as BMI and age. Since the BMI was calculated based on self-reported measures, to correct for potential biases associated with self-reported height and weight, regression equations were used (described in the methods section).

## Conclusions

The study highlights that young Polish females living in more affluent regions or from more affluent families more frequently consume fruit and vegetables, being a high-cost food. In contrast, females from economically deprived regions are more likely to present unhealthy dietary behaviours with frequent consumption of fast-foods and sweets. The results of our study exposed the regional discrepancies in dietary intake within the same country, suggesting that the national public health interventions focused on education alone may not bring the expected outcomes [40]. In fact, allocating equal funds for promoting fruit and vegetable intake in affluent regions might be somewhat wasteful. Recognising geographical distribution of dietary patterns within the country and shifting the resources to economically disadvantaged regions could be a more efficient strategy. More importantly, region-specific interventions that aim to increase fruit and vegetables consumption among adolescent girls and young females should place a strong focus on increasing the affordability and access to healthy foods in the most economically deprived regions. The study proposes that the analysis of region’s Gross Domestic Product can be a simple and inexpensive strategy that can be used for a preliminary identification of populations with unhealthy dietary behaviours.

## Supplementary information

**Additional file 1: Figure S1*.*** Flow chart of sample collection. **Figure S2.** Visual representation of the regional distribution of dietary patterns: odds ratios adjusted for age (years) and BMI (as categorical variable according to IOTF standards [37); for girls 13–18 years old according to age-sex-specific BMI cut-offs; for girls > 18 years old according to cut-offs for girls at age 18). **Table S1.** Characteristics of Poland by regions, based on the national statistical office data (GUS). **Table S2***.* Sample distribution (%) in relation to family socioeconomic status (SES) and its single factors by country regions. **Table S3.** Factor-loading matrix for the 4 major dietary patterns identified by principal component analysis. **Table S4**. Sample characteristics by age and weight status (mean and 95% confidence interval or % of the sample). **Table S5**. Adjusted odds ratios (95% Confidence intervals) for single factors of family socioeconomic status by tertiles of dietary patterns. **Table S6.** Crude odd ratios (95% confidence intervals) for country region and family socioeconomic status (SES), and its single factors, by tertiles of dietary patterns.

## Data Availability

The authors confirm that the data supporting the findings of this study are available within the article and its supplementary materials.
