# Supplementary material for: Regional and socioeconomic variations in dietary patterns in a representative sample of young polish females: a cross-sectional study (GEBaHealth project)
Source: Nutr J. 2020 Apr 3;19:26. doi: 10.1186/s12937-020-00546-8 (PMC7126359; doi:10.1186/s12937-020-00546-8)
Supplement: Supplementary file 1 — Additional file 1: Figure S1. Flow chart of sample collection. Figure S2. Visual representation of the regional distribution of dietary patterns: odds ratios adjusted for age (years) and BMI (as categorical variable according to IOTF standards [37); for girls 13–18 years old according to age-sex-specific BMI cut-offs; for girls > 18 years old according to cut-offs for girls at age 18). Table S1. Characteristics of Poland by regions, based on the national statistical office data (GUS). Table S2. Sample distribution (%) in relation to family socioeconomic status (SES) and its single factors by country regions. Table S3. Factor-loading matrix for the 4 major dietary patterns identified by principal component analysis. Table S4. Sample characteristics by age and weight status (mean and 95% confidence interval or % of the sample). Table S5. Adjusted odds ratios (95% Confidence intervals) for single factors of family socioeconomic status by tertiles of dietary patterns. Table S6. Crude odd ratios (95% confidence intervals) for country region and family socioeconomic status (SES), and its single factors, by tertiles of dietary patterns. [file 12937_2020_546_MOESM1_ESM.docx]

**Supplementary data**


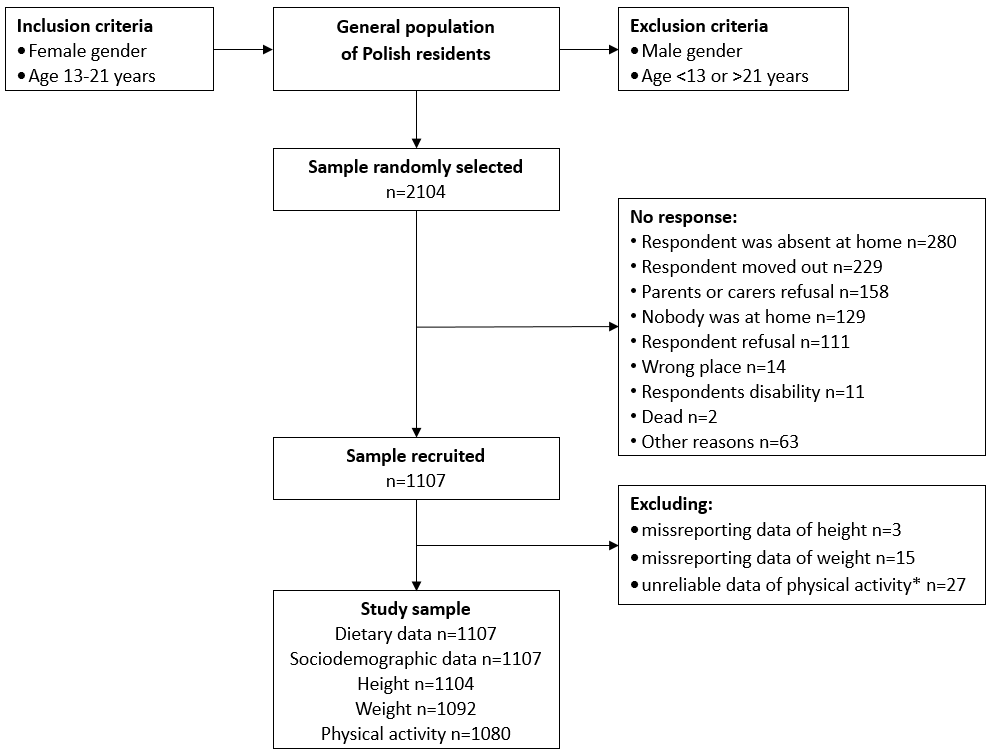


**Figure S1**. Flow chart of sample collection. Notes: *mean weekly time of activity >960 minutes/day.

**
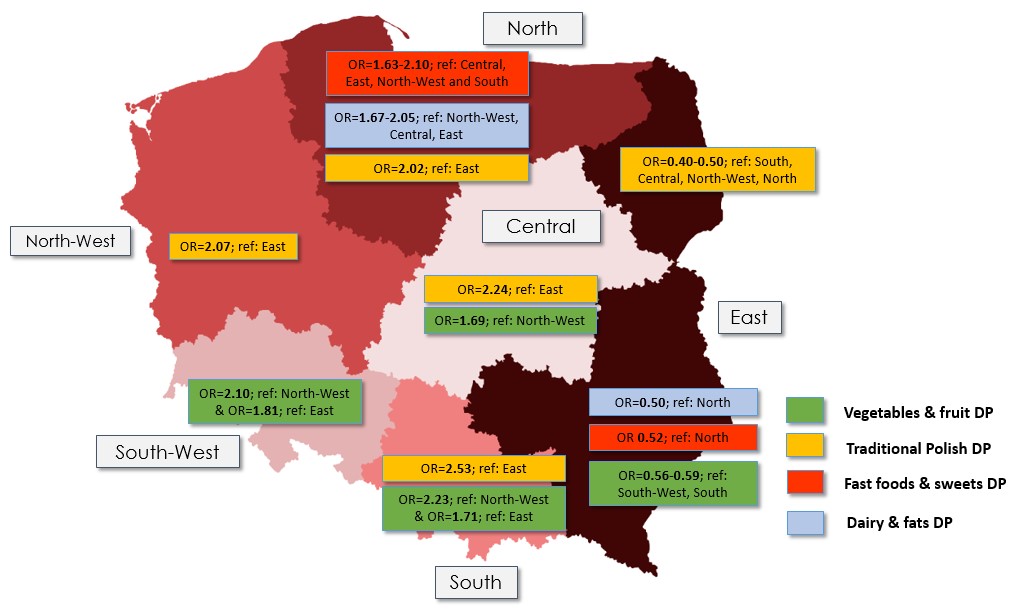
**

**Figure S2.** Visual representation of the regional distribution of dietary patterns: odds ratios adjusted for age (years) and BMI (as categorical variable according to IOTF standards [37); for girls 13-18 years old according to age-sex-specific BMI cut-offs; for girls >18 years old according to cut-offs for girls at age 18)

**Table S1**. Characteristics of Poland by regions, based on the national statistical office data (GUS)

| Variables | Poland | East | North | North-West | South | South-West | Central |
| --- | --- | --- | --- | --- | --- | --- | --- |
| **General population** ^a^ |  |  |  |  |  |  |  |
| Gross Domestic Product (%) | 100 | 69.7 | 84.8 | 95.1 | 98.8 | 104.8 | 140.4 |
| Average monthly per capita expenditures of households (%) | | | | |  |  |  |
| *On food and non-alcoholic beverages* | 26.0 | 29.1 | 26.8 | 27.0 | 25.9 | 25.6 | 23.2 |
| *On recreation and culture* | 6.8 | 6.1 | 6.4 | 6.1 | 6.7 | 6.9 | 8.0 |
| Average monthly gross wages and salaries (%) | 100 | 87.3 | 91.1 | 90.0 | 99.2 | 97.8 | 116.5 |
| Unemployment rate (%) | 10.3 | 12.0 | 11.2 | 9.3 | 10.2 | 10.8 | 9.1 |
| Life expectancy (years) ^b^ |  |  |  |  |  |  |  |
| *Males* | 73.8 | 74.2 | 73.6 | 73.5 | 74.4 | 73.9 | 73.0 |
| *Females* | 81.7 | 82.6 | 81.5 | 81.1 | 81.9 | 81.4 | 81.4 |
| **Females aged 15-29 year** ^c^ | | | |  |  |  |  |
| Self-reported health (%) | | | |  |  |  |  |
| *Good or very good* | 89.1 | 91.0 | 87.8 | 87.8 | 88.9 | 90.1 | 87.9 |
| *Below good* | 10.9 | 9.1 | 12.2 | 12.2 | 11.1 | 10.0 | 12.1 |
| Chronic diseases occurrence (%) | 31.0 | 22.2 | 35.7 | 32.1 | 32.3 | 30.4 | 33.7 |
| Body Mass Index ^#^ (%) |  |  |  |  |  |  |  |
| *Underweight* | 11.9 | 12.0 | 11.1 | 9.5 | 14.3 | 13.2 | 12.5 |
| *Normal weight* | 72.0 | 73.6 | 71.8 | 71.1 | 72.6 | 70.5 | 69.8 |
| *Overweight and obese* | 16.2 | 14.5 | 17.1 | 19.4 | 13.2 | 16.3 | 17.7 |
| Smoking (%) | 16.0 | 13.3 | 20.3 | 16.7 | 15.1 | 17.7 | 15.4 |
| Alcohol consumption at least once a week (%) | 10.0 | 7.9 | 11.1 | 9.8 | 10.1 | 12.2 | 10.4 |

Notes: data extracted from National Statistical Office (GUS): ^a^  Regions of Poland (GUS, 2014) [24], ^b^ Expectancy tables of Poland in 2018 (GUS, 2019) [25] ^c^ Health status of population in Poland in 2014 (GUS, 2016) [26]. ^#^  BMI was based on self-reported data and was categorised according to WHO criateria for adults as follows: < 18.50 kg/m^2^(underweight), 18.50–24.99 kg/m^2^ (normal weight), 25.00-29.99 kg/m^2^ (overweight), and ≥ 30 kg/m^2^ (obesity) [37].

**Table S2.** Sample distribution (%) in relation to family socioeconomic status (SES) and its single factors by country regions ^a^

| Variables | Total | East | North | North-West | South | South-West | Central | *P* |
| --- | --- | --- | --- | --- | --- | --- | --- | --- |
| Number of respondents | 1107 | 200 | 164 | 182 | 236 | 106 | 218 |  |
| SES index ^b^ |  |  |  |  |  |  |  | 0.0740 |
| low | 36.2 | 39.9 | 34.0 | 37.6 | 38.7 | 31.0 | 33.0 |  |
| average | 30.7 | 28.1 | 38.5 | 31.5 | 24.8 | 39.3 | 28.6 |  |
| high | 33.2 | 32.0 | 27.6 | 30.9 | 36.5 | 29.7 | 38.4 |  |
| Components of SES index |  |  |  |  |  |  |  |  |
| Mother’s education |  |  |  |  |  |  |  | 0.2386 |
| primary/lower secondary | 39.8 | 37.2 | 43.5 | 43.1 | 39.5 | 46.9 | 33.5 |  |
| upper secondary | 43.8 | 44.1 | 45.3 | 40.1 | 45.5 | 38.3 | 46.1 |  |
| higher | 16.5 | 18.7 | 11.2 | 16.8 | 15.0 | 14.8 | 20.4 |  |
| Father’s education |  |  |  |  |  |  |  | 0.0004 |
| primary/lower secondary | 52.4 | 51.5 | 53.7 | 58.1 | 51.1 | 59.0 | 45.5 |  |
| upper secondary | 35.1 | 40.7 | 39.6 | 28.5 | 36.9 | 28.8 | 33.3 |  |
| higher | 12.5 | 7.8 | 6.7 | 13.4 | 12.0 | 12.1 | 21.2 |  |
| Economic status |  |  |  |  |  |  |  | 0.0210 |
| below average | 3.9 | 5.1 | 3.6 | 1.7 | 4.8 | 5.7 | 3.2 |  |
| average | 80.0 | 83.0 | 82.6 | 87.8 | 76.4 | 70.7 | 77.0 |  |
| above average | 16.1 | 11.9 | 13.8 | 10.6 | 18.7 | 23.5 | 19.9 |  |
| Description of household – we live: |  |  |  |  |  |  |  | 0.0814 |
| modestly ^c^ | 8.2 | 7.8 | 9.8 | 6.0 | 12.0 | 6.8 | 5.9 |  |
| relatively thriftily | 49.6 | 53.3 | 49.4 | 56.1 | 41.9 | 43.9 | 51.8 |  |
| well ^d^ | 42.2 | 38.9 | 40.8 | 37.9 | 46.1 | 49.2 | 42.3 |  |

Notes: Sample size may vary in each variables due to missing data; All data adjusted for sample weights; ^a^ Regions of Poland ranked by Gross Domestic Product (GDP) from less to more wealthy: East GDP=69.7, North GDP=84.8, North-West GDP=95.1, South GDP=98.8, South-West GDP=104.8, Central GDP=140.4, Poland GDP=100 [24] ^b^ SES index categories based on tertile distribution; ^c^ ‘Modestly’ category was developed by combining two answers: ‘we live poorly’ and ‘we live modestly’;
^d^ ‘Well’ category was developed by combining two answers: ‘we live well’ and ‘we live very well’; *P* – significance level of the chi^2^ test.

**Table S3.** Factor-loading matrix for the 4 major dietary patterns identified by principal component analysis.

| Dietary characteristics | Factor 1 | Factor 2 | Factor 3 | Factor 4 |
| --- | --- | --- | --- | --- |
|  | ‘Traditional Polish’ | ‘Fruit and vegetables’ | ‘Fast food and sweets’ | ‘Dairy and fats’ |
| Food frequency consumption of ^a^: |  |  |  |  |
| White bread (including biscuits, muffins) | 0.65 |  |  |  |
| Potatoes | 0.52 |  |  |  |
| Red meats | 0.51 |  |  |  |
| Margarine or butter | 0.45 |  |  | 0.45 |
| Fried chicken | 0.42 |  |  |  |
| Wholegrain bread | -0.48 |  |  |  |
| Green salad |  | 0.57 |  |  |
| Fruit (without juices) |  | 0.55 |  |  |
| Prepared vegetables |  | 0.55 |  |  |
| Beans |  | 0.45 |  |  |
| French fries or potato chips or corn chips or popcorn |  |  | 0.71 |  |
| Hamburgers or cheeseburgers |  |  | 0.60 |  |
| Ice cream |  |  | 0.52 |  |
| Doughnuts or pastries or cake or cookies |  |  | 0.50 |  |
| Salad dressings or mayonnaise (not diet) |  |  | 0.42 |  |
| Cheese or cheese spread |  |  |  | 0.54 |
| Whole milk |  |  |  | 0.49 |
| Food intake variety by food groups ^b^: |  |  |  |  |
| Meats, fish and eggs | 0.60 |  |  |  |
| Fats | 0.45 |  |  | 0.43 |
| Vegetables |  | 0.60 |  |  |
| Fruit |  | 0.54 |  |  |
| Sweets and snacks |  |  | 0.47 |  |
| Cereals and potatoes |  |  |  | 0.56 |
| Dairy products |  |  |  | 0.54 |
| Eigenvalues | 4.36 | 2.39 | 1.68 | 1.44 |
| Variance explained (%)^c^ | 14.5 | 9.0 | 5.6 | 4.8 |

Notes: Factor loadings of ≤ |0.40| are not shown in the table for simplicity; Sorted by loadings from 1^st^ to 4^th^ factor; All data adjusted for sample weights; ^a^ Food frequency consumption was expressed in points (range 0-4 points); ^b^ Food intake variety was expressed in foods consumed per week (with ranges from 0-4 to 0-14 foods/week); ^c^ Total variance in dietary variables explained by 4 dietary patterns is 33.9%.

**Table S4.** Sample characteristics by age and weight status (mean and 95 % confidence interval or % of the sample).

| Variables | Total | East | North | North-West | South | South-West | Central | *P* |
| --- | --- | --- | --- | --- | --- | --- | --- | --- |
| Number of participants | 1107 | 200 | 165 | 182 | 236 | 106 | 218 |  |
| Age (years) | 17.3  (17.1-17.4) | 17.2  (16.9-17.6) | 17.2  (16.8-17.6) | 17.3  (16.9-17.7) | 17.3  (17.0-17.6) | 17.2  (16.7-17.7) | 17.3  (17.0-17.7) | 0.8750 |
| BMI (kg/m^2^) | 21.0  (20.9-21.2) | 20.7  (20.3-21.1) | 20.9  (20.5-21.4) | 21.6  (21.1-22.0) | 20.9  (20.5-21.2) | 21.5  (20.8-22.2) | 21.0  (20.7-21.4) | 0.0574 |
| BMI categories ^#^ |  |  |  |  |  |  |  |  |
| thinnest grade 3 | 0 | 0 | 0 | 0 | 0 | 0 | 0 | 0.0828 |
| thinnest grade 2 | 0.5 | 0.9 | 0.0 | 1.1 | 0.7 | 0.0 | 0.0 |  |
| thinnest grade 1 | 9.7 | 11.8 | 12.4 ^a^ | 6.1 ^a^ | 8.9 | 9.5 | 9.6 |  |
| normal weight | 77.7 | 80.8 | 73.8 | 76.2 | 79.2 | 72.7 | 80.2 |  |
| overweight | 10.5 | 5.3 ^b,c,d^ | 13.5 ^b^ | 13.8 ^c^ | 10.2 | 13.4 ^d^ | 9.1 |  |
| obesity | 1.6 | 1.2 | 0.3 ^e^ | 2.9 | 1.1 | 4.4 ^e^ | 1.1 |  |

Notes: Sample size may vary in each variables due to missing data; All data adjusted for sample weights; ^#^ BMI was categorised according to IOTF standards [37], for girls 13-18 years old according to age-sex-specific BMI cut-offs, for girls >18 years old according to cut-offs for girls at age 18) [37] as follows: < 16.0 kg/m^2^ (thinnest grade 3), 16.0–16.9 kg/m^2^ (thinnest grade 2), 17.0–18.4 kg/m^2^ (thinnest grade 1), 18.5–24.9 kg/m^2^ (normal weight), 25.0–29.9 kg/m^2^ (overweight) and ≥ 30 kg/m^2^ (obesity). *P* – significance level of the Kruskal-Wallis’ test (for means) or chi^2^ test (for percentage distribution). ^a-a, b-b, …, e-e^ – signifficant differences in pairs of the sample precentages.

**Table S5.** Adjusted ^a^ odds ratios (95% Confidence intervals) for single factors of family socioeconomic status by tertiles of dietary patterns.

| Variables | Bottom tertile | ‘Traditional Polish’ | | ‘Fruit and vegetables’ | | ‘Fast food and sweets’ | | ‘Dairy and fats’ | |
| --- | --- | --- | --- | --- | --- | --- | --- | --- | --- |
|  |  | Upper tertile | *P* | Upper tertile | *P* | Upper tertile | *P* | Upper tertile | *P* |
| Mother’s education |  |  |  |  |  |  |  |  |  |
| primary/lower secondary | Ref. | 1.00 |  | 1.00 |  | 1.00 |  | 1.00 |  |
| upper secondary |  | **0.52** (0.38-0.70) | <0.0001 | **1.36** (1.01-1.83) | 0.0447 | **0.62** (0.46-0.84) | 0.0020 | 1.22 (0.90-1.65) | 0.1948 |
| higher |  | **0.24** (0.15-0.36) | <0.0001 | **1.78** (1.18-2.68) | 0.0060 | **0.65** (0.43-0.98) | 0.0375 | **0.53** (0.35-0.81) | 0.0028 |
| Father’s education |  |  |  |  |  |  |  |  |  |
| primary/lower secondary | Ref. | 1.00 |  | 1.00 |  | 1.00 |  | 1.00 |  |
| upper secondary |  | **0.50** (0.37-0.67) | <0.0001 | **1.87** (1.38-2.53) | <0.0001 | **0.66** (0.49-0.89) | 0.0060 | 1.14 (0.84-1.54) | 0.3986 |
| higher |  | **0.27** (0.17-0.43) | <0.0001 | **2.96** (1.86-4.71) | <0.0001 | **0.55** (0.36-0.86) | 0.0087 | **0.62** (0.41-0.96) | 0.0309 |
| Economic status |  |  |  |  |  |  |  |  |  |
| below average | Ref. | 1.00 |  | 1.00 |  | 1.00 |  | 1.00 |  |
| average |  | **0.44** (0.20-0.93) | 0.0319 | 1.85 (0.94-3.64) | 0.0735 | 0.92 (0.44-1.92) | 0.8285 | 1.57 (0.72-3.42) | 0.2531 |
| above average |  | **0.29** (0.12-0.66) | 0.0032 | 2.11 (0.99-4.50) | 0.0525 | 1.28 (0.59-2.81) | 0.5272 | 1.09 (0.47-2.52) | 0.8367 |
| Description of household – we live: |  |  |  |  |  |  |  |  |  |
| modestly ^b^ | Ref. | 1.00 |  | 1.00 |  | 1.00 |  | 1.00 |  |
| relatively thriftily |  | **0.47** (0.26-0.83) | 0.0093 | 1.31 (0.80-2.16) | 0.2842 | 1.21 (0.71-2.05) | 0.4751 | 0.62 (0.37-1.04) | 0.0701 |
| well ^c^ |  | **0.27** (0.15-0.49) | <0.0001 | **1.75** (1.06-2.90) | 0.0294 | 1.60 (0.64-3.97) | 0.3133 | **0.57** (0.34-0.97) | 0.0358 |

Notes: Sample size may vary in each variables due to missing data; All data adjusted for sample weights; ^a^ Odds ratios adjusted for age (years) and BMI (as categorical variable according to IOTF standards [37]); for girls 13-18 years old according to age-sex-specific BMI cut-offs; for girls >18 years old according to cut-offs for girls at age 18); ^b^ ‘Modestly’ category was developed by combining two answers: ‘we live poorly’ and ‘we live modestly’; ^c^ ‘Well’ category was developed by combining two answers: ‘we live well’ and ‘we live very well’; *P* – significance level of the Wald’s test. Significant odds ratios are bolded.

**Table S6.** Crude odd ratios (95% confidence intervals) for country region and family socioeconomic status (SES), and its single factors, by tertiles of dietary patterns.

| Variables | Bottom tertile | ‘Traditional Polish’ | | ‘Fruit and vegetables’ | | ‘Fast food and sweets’ | | ‘Dairy and fats’ | |
| --- | --- | --- | --- | --- | --- | --- | --- | --- | --- |
|  |  | Upper tertile | *P* | Upper tertile | *P* | Upper tertile | *P* | Upper tertile | *P* |
| Region^a^ |  |  |  |  |  |  |  |  |  |
| East | Ref. | 1.00 |  | 1.00 |  | 1.00 |  | 1.00 |  |
| North |  | **1.94** (1.21-3.09) | 0.0053 | 1.41 (0.89-2.24) | 0.1442 | **1.79** (1.12-2.87) | 0.0146 | **1.86** (1.16-2.99) | 0.0099 |
| North-West |  | **2.11** (1.32-3.36) | 0.0017 | 0.87 (0.54-1.38) | 0.5444 | 0.91 (0.58-1.44) | 0.6911 | 1.13 (0.72-1.78) | 0.5827 |
| South |  | **2.46** (1.56-3.87) | <0.0001 | **1.75** (1.13-2.71) | 0.0118 | 0.89 (0.58-1.36) | 0.5791 | 1.38 (0.90-2.11) | 0.1427 |
| South-West |  | 1.58 (0.93-2.70) | 0.0914 | **1.87** (1.11-3.14) | 0.0179 | 1.10 (0.65-1.87) | 0.7197 | 1.48 (0.87-2.52) | 0.1414 |
| Central |  | **2.06** (1.31-3.25) | 0.0017 | 1.55 (0.99-2.40) | 0.0520 | 1.08 (0.70-1.66) | 0.7374 | 1.07 (0.69-1.68) | 0.7557 |
| North | Ref. | 1.00 |  | 1.00 |  | 1.00 |  | 1.00 |  |
| East |  | **0.52** (0.32-0.82) | 0.0053 | 0.71 (0.45-1.13) | 0.1441 | **0.56** (0.35-0.89) | 0.0146 | **0.54** (0.33-0.86) | 0.0099 |
| North-West |  | 1.09 (0.68-1.73) | 0.7296 | 0.61 (0.38-1.00) | 0.0493 | **0.51** (0.31-0.83) | 0.0066 | **0.61** (0.37-0.99) | 0.0463 |
| South |  | 1.27 (0.81-2.00) | 0.3010 | 1.24 (0.78-1.96) | 0.3545 | **0.49** (0.31-0.78) | 0.0027 | 0.74 (0.46-1.18) | 0.2031 |
| South-West |  | 0.82 (0.48-1.39) | 0.4529 | 1.32 (0.77-2.26) | 0.3038 | 0.61 (0.35-1.08) | 0.0869 | 0.80 (0.45-1.40) | 0.4269 |
| Central |  | 1.06 (0.67-1.68) | 0.7872 | 1.10 (0.69-1.73) | 0.6923 | **0.60** (0.37-0.96) | 0.0333 | **0.54** (0.34-0.86) | 0.0100 |
| North-West | Ref. | 1.00 |  | 1.00 |  | 1.00 |  | 1.00 |  |
| East |  | **0.48** (0.30-0.76) | 0.0017 | 1.16 (0.72-1.85) | 0.5442 | 1.10 (0.69-1.73) | 0.6926 | 0.88 (0.56-1.39) | 0.5866 |
| North |  | 0.92 (0.58-1.47) | 0.7293 | 1.63 (1.00-2.66) | 0.0493 | **1.97** (1.20-3.21) | 0.0066 | **1.64** (1.01-2.68) | 0.0463 |
| South |  | 1.17 (0.75-1.83) | 0.4958 | **2.02** (1.27-3.21) | 0.0028 | 0.97 (0.62-1.52) | 0.8976 | 1.21 (0.78-1.89) | 0.3934 |
| South-West |  | 0.75 (0.44-1.28) | 0.2918 | **2.16** (1.25-3.71) | 0.0052 | 1.21 (0.70-2.09) | 0.4980 | 1.31 (0.76-2.25) | 0.3287 |
| Central |  | 0.98 (0.62-1.55) | 0.9330 | **1.79** (1.12-2.85) | 0.0147 | 1.18 (0.75-1.86) | 0.4754 | 0.88 (0.56-1.40) | 0.5944 |
| South | Ref. | 1.00 |  | 1.00 |  | 1.00 |  | 1.00 |  |
| East |  | **0.41** (0.26-0.64) | <0.0001 | **0.57** (0.37-0.88) | 0.0117 | 1.13 (0.74-1.73) | 0.5755 | 0.73 (0.47-1.12) | 0.1428 |
| North |  | 0.79 (0.50-1.24) | 0.3011 | 0.81 (0.51-1.27) | 0.3548 | **2.02** (1.27-3.21) | 0.0027 | 1.35 (0.85-2.16) | 0.2026 |
| North-West |  | 0.86 (0.54-1.34) | 0.4977 | **0.49** (0.31-0.79) | 0.0028 | 1.03 (0.66-1.61) | 0.8968 | 0.82 (0.53-1.29) | 0.3931 |
| South-West |  | 0.64 (0.38-1.08) | 0.0955 | 1.07 (0.64-1.78) | 0.8030 | 1.24 (0.74-2.10) | 0.4126 | 1.08 (0.64-1.82) | 0.7750 |
| Central |  | 0.84 (0.54-1.30) | 0.4319 | 0.88 (0.57-1.37) | 0.5777 | 1.22 (0.79-1.86) | 0.3699 | 0.73 (0.48-1.12) | 0.1441 |
| South-West | Ref. | 1.00 |  | 1.00 |  | 1.00 |  | 1.00 |  |
| East |  | 0.63 (0.37-1.08) | 0.0915 | **0.54** (0.32-0.90) | 0.0179 | 0.91 (0.53-1.54) | 0.7195 | 0.67 (0.40-1.14) | 0.1412 |
| North |  | 1.23 (0.72-2.09) | 0.4535 | 0.76 (0.44-1.29) | 0.3036 | 1.63 (0.93-2.85) | 0.0869 | 1.25 (0.72-2.17) | 0.4170 |
| North-West |  | 1.33 (0.78-2.27) | 0.2917 | **0.46** (0.27-0.80) | 0.0052 | 0.83 (0.48-1.43) | 0.4983 | 0.76 (0.44-1.31) | 0.3288 |
| South |  | 1.56 (0.92-2.62) | 0.0956 | 0.94 (0.56-1.57) | 0.8035 | 0.80 (0.48-1.36) | 0.4133 | 0.93 (0.55-1.56) | 0.7747 |
| Central |  | 1.31 (0.77-2.21) | 0.3171 | 0.83 (0.49-1.39) | 0.4733 | 0.98 (0.57-1.68) | 0.9335 | 0.68 (0.40-1.14) | 0.1427 |
| Central | Ref. | 1.00 |  | 1.00 |  | 1.00 |  | 1.00 |  |
| East |  | **0.48** (0.31-0.76) | 0.0017 | 0.65 (0.42-1.01) | 0.0521 | 0.93 (0.60-1.44) | 0.7393 | 0.93 (0.59-1.46) | 0.7557 |
| North |  | 0.94 (0.60-1.48) | 0.7871 | 0.91 (0.58-1.45) | 0.6956 | **1.67** (1.04-2.67) | 0.0333 | **1.86** (1.16-2.98) | 0.0100 |
| North-West |  | 1.02 (0.64-1.63) | 0.9343 | **0.56** (0.35-0.89) | 0.0147 | 0.85 (0.54-1.34) | 0.4756 | 1.13 (0.72-1.77) | 0.5879 |
| South |  | 1.25 (0.80-1.95) | 0.3370 | 1.13 (0.77-1.67) | 0.5321 | 0.82 (0.54-1.26) | 0.3693 | 1.37 (0.90-2.10) | 0.1440 |
| South-West |  | 0.77 (0.45-1.29) | 0.3165 | 1.21 (0.72-2.03) | 0.4734 | 1.02 (0.60-1.74) | 0.9320 | 1.48 (0.87-2.51) | 0.1426 |
| SES index ^b^ |  |  |  |  |  |  |  |  |  |
| low | Ref. | 1.00 |  | 1.00 |  | 1.00 |  | 1.00 |  |
| average |  | **0.60** (0.43-0.84) | 0.0027 | **1.81** (1.30-2.52) | 0.0004 | 0.89 (0.64-1.24) | 0.5054 | 1.26 (0.90-1.75) | 0.1778 |
| high |  | **0.28** (0.20-0.39) | <0.0001 | **1.97** (1.42-2.74) | <0.0001 | 0.95 (0.69-1.32) | 0.7717 | 0.92 (0.67-1.26) | 0.6016 |
| Mother’s education |  |  |  |  |  |  |  |  |  |
| primary/lower secondary | Ref. | 1.00 |  | 1.00 |  | 1.00 |  | 1.00 |  |
| upper secondary |  | **0.52** (0.39-0.70) | <0.0001 | 1.34 (1.00-1.80) | 0.0523 | **0.64** (0.48-0.87) | 0.0036 | 1.22 (0.91-1.63) | 0.1911 |
| higher |  | **0.24** (0.15-0.36) | <0.0001 | **1.72** (1.15-2.58) | 0.0079 | 0.67 (0.45-1.00) | 0.0513 | **0.59** (0.40-0.88) | 0.0101 |
| Father’s education |  |  |  |  |  |  |  |  |  |
| primary/lower secondary | Ref. | 1.00 |  | 1.00 |  | 1.00 |  | 1.00 |  |
| upper secondary |  | **0.49** (0.37-0.66) | <0.0001 | **1.79** (1.33-2.41) | 0.0001 | **0.67** (0.50-0.90) | 0.0070 | 1.18 (0.88-1.58) | 0.2716 |
| higher |  | **0.26** (0.16-0.42) | <0.0001 | **2.95** (1.86-4.68) | <0.0001 | **0.58** (0.38-0.89) | 0.0127 | **0.66** (0.43-1.00) | 0.0476 |
| Economic status |  |  |  |  |  |  |  |  |  |
| below average | Ref. | 1.00 |  | 1.00 |  | 1.00 |  | 1.00 |  |
| average |  | **0.43** (0.20-0.91) | 0.0278 | 1.74 (0.91-3.35) | 0.0954 | 0.86 (0.43-1.75) | 0.6855 | 1.46 (0.68-3.14) | 0.3344 |
| above average |  | **0.29** (0.13-0.65) | 0.0026 | 1.95 (0.93-4.09) | 0.0729 | 1.26 (0.58-2.75) | 0.5535 | 1.10 (0.48-2.52) | 0.8217 |
| Description of household – we live: |  |  |  |  |  |  |  |  |  |
| modestly ^c^ | Ref. | 1.00 |  | 1.00 |  | 1.00 |  | 1.00 |  |
| relatively thriftily |  | **0.45** (0.25-0.80) | 0.0066 | 1.29 (0.79-2.09) | 0.3117 | 1.20 (0.72-2.02) | 0.4806 | 0.61 (0.37-1.02) | 0.0597 |
| well ^d^ |  | **0.28** (0.16-0.51) | <0.0001 | 1.63 (1.00-2.68) | 0.0514 | 1.58 (0.93-2.66) | 0.0872 | **0.59** (0.35-0.99) | 0.0431 |

Notes: Sample size may vary in each variables due to missing data; All data adjusted for sample weights; ^a^ Regions of Poland ranked by Gross Domestic Product (GDP) from less to more wealthy: East GDP=69.7, North GDP=84.8, North-West GDP=95.1, South GDP=98.8, South-West GDP=104.8, Central GDP=140.4, Poland GDP=100 [24]; ^b^ SES index categories based on tertile distribution; ^c^ ‘Modestly’ category was developed by combining two answers: ‘we live poorly’ and ‘we live modestly’; ^d^ ‘Well’ category was developed by combining two answers: ‘we live well’ and ‘we live very well’; *P* – significance level of the Wald’s test. Significant odds ratios are bolded.
